# Nuclear Speckle RNA Binding Proteins Remodel Alternative Splicing and the Non-coding Arabidopsis Transcriptome to Regulate a Cross-Talk Between Auxin and Immune Responses

**DOI:** 10.3389/fpls.2018.01209

**Published:** 2018-08-21

**Authors:** Jérémie Bazin, Natali Romero, Richard Rigo, Celine Charon, Thomas Blein, Federico Ariel, Martin Crespi

**Affiliations:** ^1^CNRS, INRA, Institute of Plant Sciences Paris-Saclay IPS2, Univ Paris Sud, Univ Evry, Univ Paris-Diderot, Sorbonne Paris-Cite, Universite Paris-Saclay, Orsay, France; ^2^Instituto de Agrobiotecnologıa del Litoral, CONICET, Universidad Nacional del Litoral, Santa Fe, Argentina

**Keywords:** RNA binding proteins, RNP complexes, alternative splicing, immune response, auxin

## Abstract

Nuclear speckle RNA binding proteins (NSRs) act as regulators of alternative splicing (AS) and auxin-regulated developmental processes such as lateral root formation in *Arabidopsis thaliana*. These proteins were shown to interact with specific alternatively spliced mRNA targets and at least with one structured lncRNA, named *Alternative Splicing Competitor* RNA. Here, we used genome-wide analysis of RNAseq to monitor the NSR global role on multiple tiers of gene expression, including RNA processing and AS. NSRs affect AS of 100s of genes as well as the abundance of lncRNAs particularly in response to auxin. Among them, the *FPA* floral regulator displayed alternative polyadenylation and differential expression of antisense *COOLAIR* lncRNAs in *nsra/b* mutants. This may explains the early flowering phenotype observed in *nsra* and *nsra/b* mutants. GO enrichment analysis of affected lines revealed a novel link of NSRs with the immune response pathway. A RIP-seq approach on an NSRa fusion protein in mutant background identified that lncRNAs are privileged direct targets of NSRs in addition to specific AS mRNAs. The interplay of lncRNAs and AS mRNAs in NSR-containing complexes may control the crosstalk between auxin and the immune response pathway.

## Introduction

RNA binding proteins (RBPs) have been shown to affect all steps of post-transcriptional gene expression control, including alternative splicing (AS), silencing, RNA decay, and translational control (Bailey-Serres et al., 2009). The *Arabidopsis thaliana* genome encodes for more than 200 proteins predicted to bind RNAs. The picture becomes even more complex since over 500 proteins were found to bind polyA+ RNA in a recent study attempting to define the RNA interactome using affinity capture and proteomics (Marondedze et al., 2016). However, only a small subset of RBPs has been functionally assigned in plants. The versatility of RBPs on gene expression regulation has been recently highlighted by the identification of several among them acting at multiple steps of post-transcriptional gene regulation (Lee and Kang, 2016; Oliveira et al., 2017). During mRNA maturation, the transcript acquires a complex of proteins at each exon–exon junction during pre-mRNA splicing that influences the subsequent steps of mRNA translation and decay (Maquat, 2004). Although all RBPs bind RNA, they exhibit different RNA-sequence specificities and affinities. As a result, cells are able to generate diverse ribonucleoprotein complexes (RNPs) whose composition is unique to each mRNA and these complexes are further remodeled during the life of the mRNA in order to determine its fate. One approach to determine RBP function consisted in the identification of all interacting molecules (the so-called RNPome) of a specific RNP and the conditions of their association. The ribonucleoprotein immunopurification assay facilitates the identification and quantitative comparison of RNA association to specific proteins under different experimental conditions. This approach has been successfully used to elucidate the genome-wide role of a number of plant RBPs involved in pre-mRNA splicing, stress granule formation or translational control (Sorenson and Bailey-Serres, 2014; Gagliardi and Matarazzo, 2016; Foley et al., 2017; Köster and Meyer, 2018).

The nuclear speckle RNA binding proteins (NSRs) are a family of RBPs that act as regulators of AS and auxin regulated developmental processes such as lateral root formation in *Arabidopsis thaliana*. These proteins were shown to interact with some of their alternatively spliced mRNA targets and at least with one structured lncRNA, named *Alternative Splicing Competitor* RNA (*ASCO*) (Bardou et al., 2014). Overexpression of *ASCO* was shown to affect AS of a subset of mRNA regulated by NSRs, similar to *nsra/b* double mutants, and *ASCO* was also shown to compete *in vitro* with the binding of one AS mRNA target. This study suggested that plant lncRNAs are able to modulate AS of mRNA by hijacking RBPs, such as NSRs, involved in splicing (Romero-Barrios et al., 2018). In addition, transcriptome analysis using microarrays and specific AS analysis on a subset of mRNAs suggested a role of NSR in transcriptome remodeling in response to auxin (Bardou et al., 2014).

Here we used genome wide analysis to monitor the NSR global role on multiple tiers of gene expression, including RNA processing and AS. This allowed us to find a new role of NSR in the control of flowering time regulators as well as to suggest that NSRs control the crosstalk between auxin and the immune response pathway.

## Results

### Auxin Regulation of Gene Expression Is Altered in *nsra/b* Double Mutant

**FIGURE 1:**
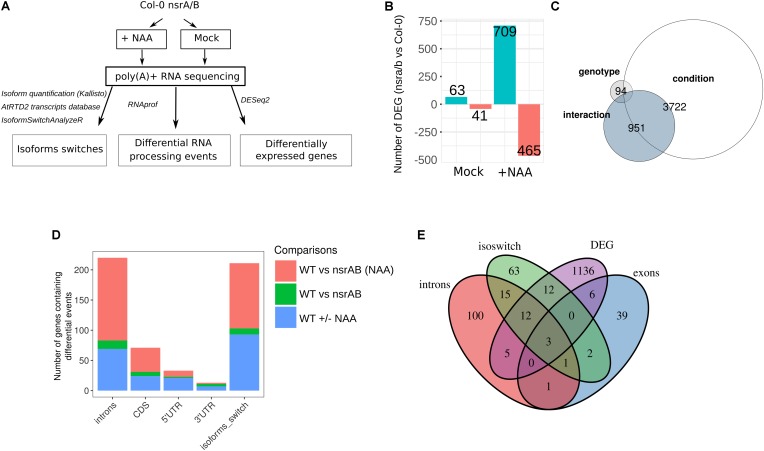
The *nsra/b* mutant shows changes in auxin-dependent gene expression and AS. **(A)** Experimental design to analyze expression and alternative splicing (AS) changes in response to the synthetic auxin NAA in *nsra/b* compared to Col-0 (WT). **(B)** Number of up or down regulated genes between *nsra/b* and Col-0 (WT) in control and NAA treated seedlings. **(C)** Comparison of gene sets whose expression is significantly affected by the *nsra/b* mutation only (genotype), the NAA treatment (condition) or the interaction between the two factors (interaction). **(D)** Number of genes containing at least one differential RNA processing events in introns, CDS, 5′ UTR, and 3′ UTR or a switching isoform in each possible pairwise comparison. **(E)** Comparison of differentially spliced genes identified by the different methods. The exon group represents genes with a differential processing events in 5′ UTR, 3′ UTR, or CDS exons.

To characterize the role of NSRs in the control of auxin regulated gene expression, we performed paired-end strand specific RNA sequencing on the *nsra/nsrb* (*nsra/b*) double mutant and wild type (Col-0) seedlings treated for 24 h with the synthetic auxin NAA (100 nM) or a mock solution (Bardou et al., 2014; Tran et al., 2016) (**Figure 1A**).

In mock treated samples, 63 and 41 genes were found to be differentially up and down-regulated between mutant and wild type seedlings (**Supplementary Table S1B**). Remarkably, in response to auxin, we identified 709 and 465 genes significantly up and down-regulated in *nsra/b*, compared to wild type (**Figure 1B** and **Supplementary Table S1B**). Principal component analysis (PCA) showed a dispersion of the data compatible with statistical comparisons between groups (**Supplementary Figure S1**). Multifactor analysis of differential gene expression further showed that *nsra/b* mutation has a major effect on auxin-regulated gene expression. Indeed, a set of 951 genes showed significant interaction between genotype and auxin regulation (**Figure 1C** and **Supplementary Table S1B**). This is in agreement with our previous findings indicating that NSRs mediate auxin regulation of gene expression (Bardou et al., 2014).

We have previously shown that NSRs modulate auxin-induced AS of a particular subset of genes using specific qRT-PCR assays (Bardou et al., 2014). We use now our RNA-seq dataset to characterize genome-wide effects of NSRs on AS and more generally on RNA processing (**Figure 1A**). To this end, we made use of the RNAprof software, which implements a gene-level normalization procedure and can compare RNA-seq read distributions on transcriptional units to detect significant profile differences. This approach allows *de novo* identification of RNA processing events independently of any gene feature or annotation independently of gene expression differences (Tran et al., 2016). RNAprof results were parsed to retain only highly significant differential RNA processing events (p.adj < 10e-4) and further crossed with gene annotation in order to classify them according to their gene features. The majority of events overlapped with intronic regions (**Figure 1D** and **Supplementary Table S1C**), which is in accordance with data showing that intron retention is the major event of AS in plants (Ner-Gaon et al., 2004). The effect of *nsra/b* on RNA processing and splicing is enhanced in response to NAA. In other words the vast majority of differential events between *nsra/b* and wild type plants were identified essentially in presence of auxin.

To further support the results from RNAprof and to gain knowledge on the functional consequences of NSR mediated AS events, we quantified mRNA transcript isoforms of the AtRTD2 database (Brown et al., 2017) using *kallisto* (Bray et al., 2016). Then, we searched for marked changes in isoform usage using IsoformSwitchAnalyzeR package (Vitting-Seerup and Sandelin, 2017), which allows statistical detection and visualization and prediction of functional consequences of isoform switching events. As a result, we identified 118 NSR-dependent isoform switching events including 108 only detected in NAA-treated samples (**Figure 1E** and **Supplementary Table S1D**). Comparison of gene sets affected in their steady state abundance, containing differential RNA processing or isoforms switching events in *nsra/b* highlighted the fact that most differentially spliced genes are not differentially expressed. In addition, over 35% of genes predicted with isoforms switching events were also found using RNAprof (**Figure 1E**).

### NSRs Affect the Abundance of Numerous LncRNAs

**FIGURE 2:**
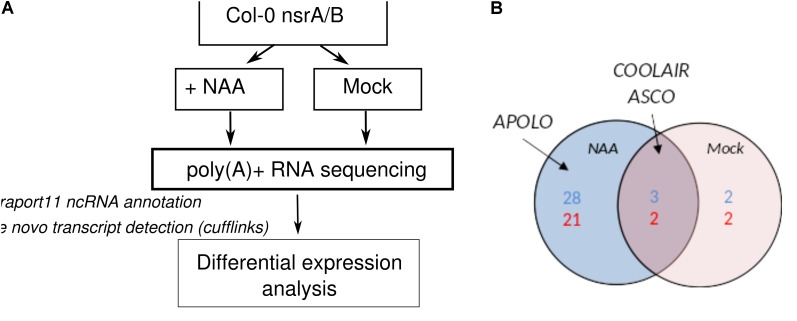
NSRs control the expression of numerous lncRNAs. **(A)** Experimental design to analyze changes in lncRNAs expression in *nsra/b* vs. Col-0 (WT) in control condition and in response to the synthetic auxin NAA. LncRNAs were predicted *de novo* using cufflinks and merged with Araport11 lncRNA annotation. **(B)** Differentially expressed antisense (blue) and intergenic (red) lncRNA in nsra/b compared to Col-0 in mock (red circle) or NAA treated (blue circle) seedlings. Already characterized lncRNA ASCO (Bardou et al., 2014), *APOLO* (Ariel et al., 2014), and COOLAIR (Liu et al., 2009) are indicated on the figure.

The activity of NSR proteins on AS is modulated by the lncRNA *ASCO* and the abundance of *ASCO* RNA is increased in *nsra/b* mutant (Bardou et al., 2014). Therefore, we conducted a global analysis of lncRNAs detection and expression in our RNA-seq datasets. Annotated lncRNA (Araport11) were combined with *de novo* predicted transcripts and further classified based on their location in intergenic and antisense regions of coding genes (**Figure 2A**). More than 2440 lncRNAs were detected in our RNAseq data with more than 1 TPM (**Supplementary Table S1A**) in at least three samples. In mock conditions, differential expression analysis served to identify five antisense and four intergenic lncRNAs differentially expressed between mutant and wild type seedlings, whereas 31 intergenic and 23 antisense lncRNAs were found to be differentially regulated between mutant and wild type in the presence of auxin (**Figure 2B**). Differentially expressed lncRNAs included a number of well-characterized lncRNAs such as *APOLO*, which as been shown to influence root gravitropism in response to auxin via its action on PINOID protein kinase expression dynamics. In addition, the expression of lncRNA *ASCO*, shown to interact with NSR to modulate AS of its mRNA targets, was also affected in in nsra/b suggesting a feedback regulation of NSR on ASCO lncRNA (**Figure 2B**).

### NSRa Is Involved in the Control Flowering Time Through the Modulation of the *COOLAIR/FLC* Module

**FIGURE 3:**
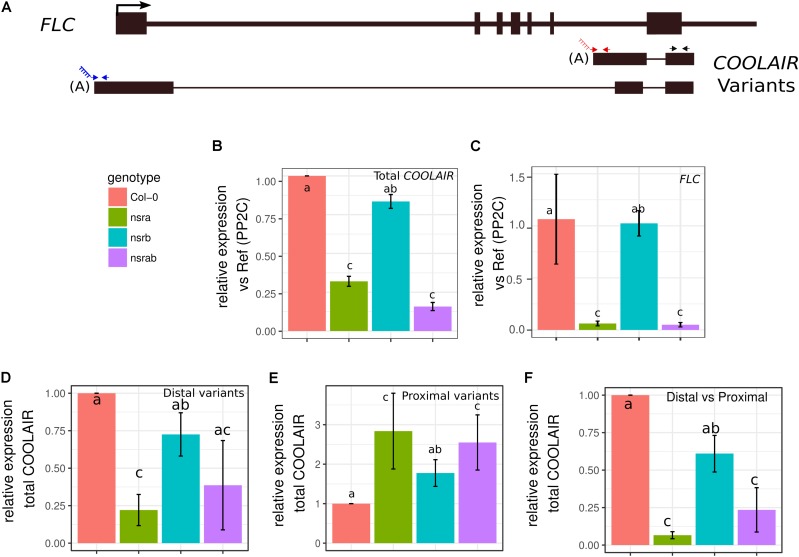
NSRs modulate the relative abundance of lncRNA *COOLAIR* variants. **(A)** Schematic representation of transcripts from the *FLC/COOLAIR* locus. *COOLAIR* isoforms are shown including positions of primers (arrows) used to measure distal (blue arrows) and proximal (red arrows) and total (black arrows) COOLAIR variant abundance. Black rectangles and black lines denote exons and introns, respectively. **(B)** *COOLAIR* and **(C)** *FLC* abundance measured by RT-qPCR in *nsra, nsrb, nsra/b* and Col-0 in seedlings. **(D)** Proximal and **(E)** distal variant usage normalized to the total amount of *COOLAIR*. **(F)** Distal vs. proximal variant usage ratio. Data represent the mean of three biological replicates ± standard error. Results were analyzed by one-way analysis of variance (ANOVA) followed by Tukey’s *post-hoc* test: groups with different letters are statistically different (*p* ≤ 0.05) and groups with the same letters are statistically equal (*p* ≤ 0.05). Significance was determined using an ANOVA coupled with a Tukey pairwise test (*p*-value < 0.05).

Interestingly, we also identified the lncRNA *COOLAIR* as down regulated in *nsra/b*, both in mock or NAA treated samples (**Figure 2B**). *COOLAIR* designate a set of transcripts expressed in antisense orientation of the locus encoding the floral repressor FLC (Whittaker and Dean, 2017). Two main classes of *COOLAIR* lncRNAs are produced by AS and polyadenylaton of antisense transcripts generated from the FLC locus. One uses a proximal splice site and a polyadenylation site located in intron 6 of *FLC*, whereas the distal one results from the use of a distal splice and polyadenylation sites located in the *FLC* promoter (reviewed in Whittaker and Dean, 2017) (**Figure 3A**).

**FIGURE 4:**
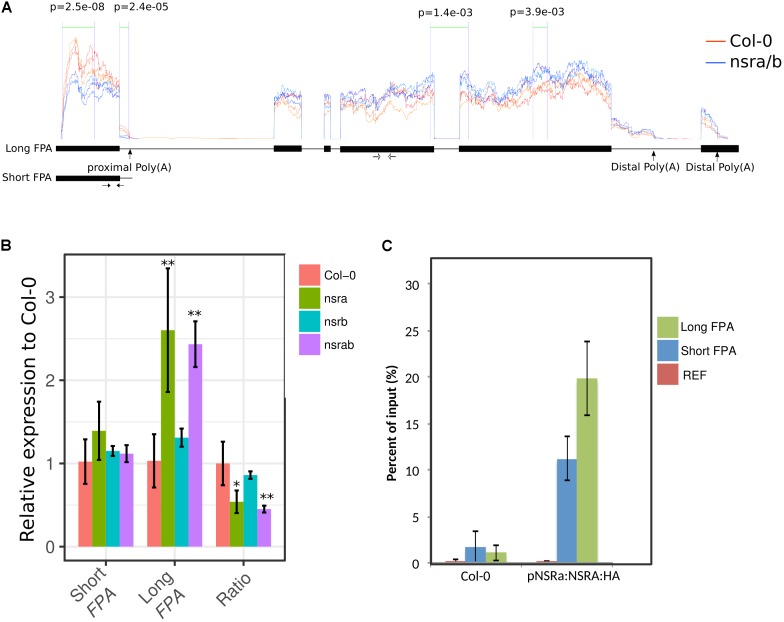
*FPA* is differentially processed in *nsra/b* plants. **(A)** The RNA processing event detected in FPA by RNAprof from the comparison of WT (in orange) and *nsra/b* (blue). Significant differential events are delimited by green lines and labeled with their *p*-value (*p*) The Y-axis show the normalized RNA-seq coverage from RNAprof. Section between two purple lines with *p*-values indicated denote significant differences between nucleotide based coverage. Orange and blue traces correspond triplicate samples of Col-0 and nsra/b treated with a mock solution, respectively. The X-axis represents gene coordinates (boxes and lines representing exons and introns, respectively). Positions of polyadenylation sites identified in Hornyik et al. (2010) are shown on the gene model as well as the two transcript variants deriving. Positions of primer pairs used to amplify the short and long *FPA* variant are indicated as black and with arrows (respectively). **(B)** Isoforms specific RT-qPCR analysis of short and long *FPA* variant and their abundance ratio in *nsra*, *nsrb*, and *nsra/b*. Depicted data is the mean of fold change compared to Col-0 ± standard deviation of three biological replicates. Significance is was determined according to a Student’s *t*-test (^∗^*p* < 0.05; ^∗∗^*p* < 0.01). **(C)** RIP assays using ProNSRa::NSRa::HA (NSRa), Col-0 (w/o: without tag) plants on total cell lysates of 10-day-old seedlings treated with 10 mM NAA for 24 h. Results of RT-qPCR are expressed as mean of the percentage of the respective INPUT signal (total signal before RIP) from three independent replicates ± standard error. Genes analyzed are a housekeeping gene (At1g13320) named here REF and FPA (AT2G43410) short and long isoforms.

**FIGURE 5:**
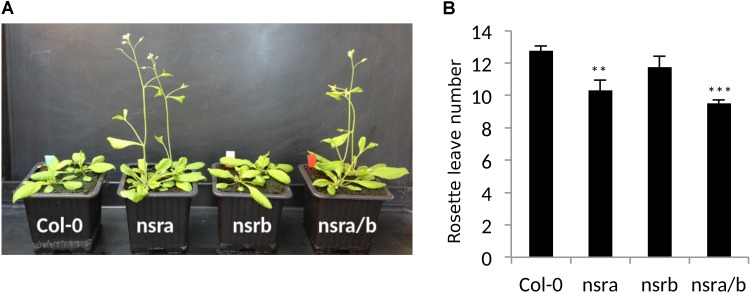
NSRa affects flowering time in Arabidopsis. **(A)** Representative picture of Col-0 *nsra*, *nsrb* and *nsra/b* at 21 days after germination. **(B)** Mean number of rosette leaves at bolting in Col-0, *nsra*, *nsrb*, and *nsra/b*. Data is mean of 12 plants ± standard deviation. Significance was determined using a Student’s *t*-test (^∗∗^*p*-value < 0.01; ^∗∗∗^*p*-value < 0.001).

Strikingly, *FLC* is one of most deregulated genes in *nsra/b* mutants in control and NAA-treated samples. Notably, it was shown that a number of splicing and RNA processing factors control *FLC* expression by modulating the ratio of *COOLAIR* proximal and distal variants (Liu et al., 2009; Marquardt et al., 2014; Whittaker and Dean, 2017). Therefore, we determined the abundance and the ratio of *COOLAIR* variants in wild type, single *nsra*, *nsrb* and the double *nsra/b* mutants in control and NAA treated conditions using a dedicated strand-specific RT-qPCR assay (Marquardt et al., 2014). First, we confirmed that total *COOLAIR* and *FLC* abundance was decreased in *nsra* and *nsra/b* but not *nsrb* (**Figures 3B,C**). More importantly, we found that relative usage of the short (proximal) variant of *COOLAIR* increased by twofold in *nsra* and *nsra/b* but not in *nsrb* leading to an increase of the ratio of distal vs. proximal *COOLAIR* isoforms in the same genotypes (**Figures 3D–F**). When analyzing the relative abundance of both variants against a housekeeping gene, we determined the decrease of total COOLAIR transcripts associated with a specific decrease of the distal variants. In contrast, proximal variant abundance remains stable (**Supplementary Figure S2**), leading to a change in relative variant usage (**Figures 3D,E**). Interestingly, the proximal *COOLAIR* variant was associated with a down-regulation of *FLC* and an early flowering phenotype (Marquardt et al., 2014). Together, these results suggest that the modulation of COOLAIR polyadenylation and/or splicing in nsra mutants contributes to the control of FLC expression. In addition, RNAprof also identified that the mRNA coding for the FPA protein (Hornyik et al., 2010) was differentially processed in *nsra/b* seedling treated with NAA (**Figure 4A**). The differential RNA processing event occurred at the end of intron 1, which has been shown to contain an alternative polyadenylation site necessary for FPA negative autoregulation (Hornyik et al., 2010). RNAprof analysis hinted a significant reduction of the short FPA variant in nsra/b mutant compared to Col-0 (**Figure 4A**). RT-qPCR analysis using isoform specific primers (**Figure 4A**) showed that the long isoform accumulated in *nsra* and *nsra/b* but not in *nsrb* whereas the short isoform remained unaffected (**Figure 4B**). Hence, our data suggested that the use of the proximal polyA site is reduced in *nsra* and *nsra/b* mutant, which is predicted to lead to an increase of the full-length functional FPA. Interestingly, FPA was shown to favor proximal *COOLAIR* variants forms (Hornyik et al., 2010), suggesting that the effect of NSR mutation on *COOLAIR* variant ratio may be mediated by changes in *FPA* polyadenylation site usage. To address this potential mechanism, we checked whether *COOLAIR* or *FPA* are direct targets of NSRa by RNA immunoprecipitation (RIP) using transgenic lines expressing a tagged version of the NSRa protein. Although we did not find *COOLAIR* binding to NSR, both the long and the short *FPA* variant were enriched in the RIP assay supporting the idea that NSRa directly influences the processing of FPA mRNA (**Figure 4C**). Given the critical role of FPA, *COOLAIR*, and FLC in flowering, we hypothesized that NSRa may be involved in the control of flowering time. Indeed, we observed that *nsra/b* mutant displays an early flowering phenotype (**Figure 5A**). We then quantified this phenotype by counting the number of rosette leaves when the flower stem emerged from the plants. Data showed that *nsra* and *nrsa/b* but not *nsrb* display an early flowering phenotype (**Figure 5B**), which is consistent with a lower expression of *FLC* in *nsra* and *nsra/b* mutants only (**Figure 4C**). Altogether, our results indicate that NSRa-dependent modulation of *FPA* polyadenylation may impacts the activity of the *COOLAIR/FLC* module, affecting flowering time in Arabidopsis.

### NSRs Affect Auxin-Dependent Expression of Biotic Stress Response Genes

**FIGURE 6:**
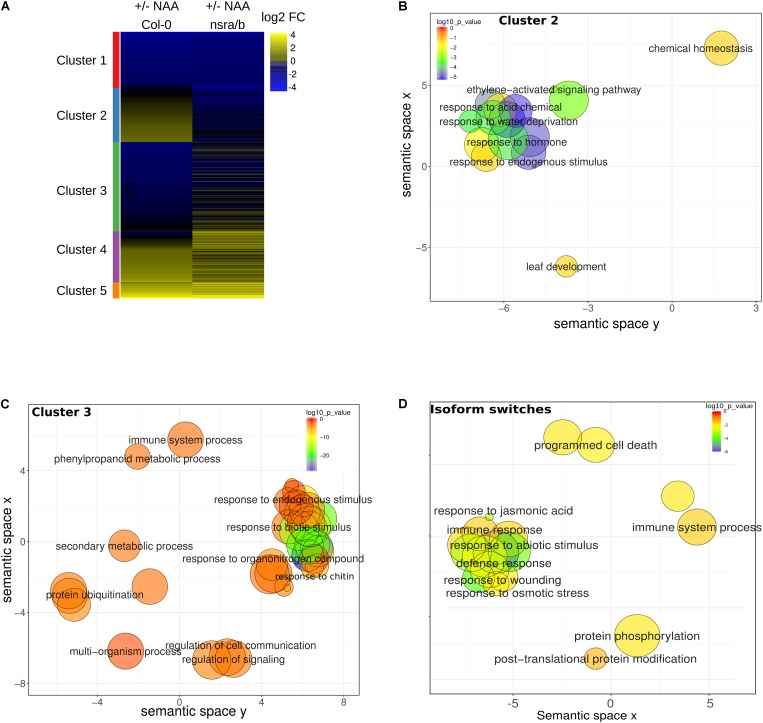
Steady state abundances and AS of genes involved in biotic stress responses are affected in nsra/b mutants. **(A)** Heatmap of log2 fold change (log2FC) expression change in response to NAA for differentially expressed genes in *nsra/b* compared to wild type. Genes were clustered using K-mean clustering, the left side bar represent the delimitation of each cluster REVIGO plots of Biological Function. **(B)** Gene Ontology (GO) of cluster 2 and **(C)** cluster 3 as defined in panel A and gene with significant isoforms switching events **(D)**. Each circle represents a significant GO category but only group with the highest significance are labeled. Related GOs have similar (x, y) coordinates.

**FIGURE 7:**
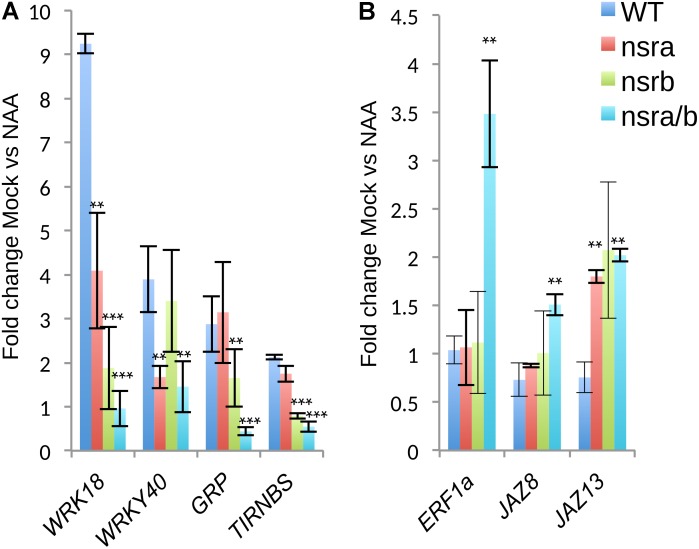
Expression analysis of a selected subset of genes by RT-qPCR. **(A)** Expression changes in response to NAA of genes belonging to cluster 2 and cluster 3 **(B)** as defined in **Figure 6A**. Expression was tested in Col-0 *nsra*, *nsrb* and *nsra/b*, on three biological replicates. Values correspond to the mean fold change of Mock treated versus NAA treated seedling of the designated genotype. Error bars correspond to ± the standard deviation of three biological replicates. Significance was determined using a Student’s *t*-test (^∗∗^*p*-value < 0.01; ^∗∗∗^*p*-value < 0.001).

To extend our understanding on the genome-wide roles of NSRs in the control of auxin-dependent gene expression, we searched the putative function of differentially expressed and/or spliced gene groups using clustering and Gene Ontology (GO) enrichment analyses. Hierarchical clustering of differentially expressed genes determine two clusters of genes showing opposite expression patterns in response to NAA in *nsra/b* as compared to wild type plants (**Figure 6A**). GO analyses revealed that cluster 2 (**Figure 6B**), e.g., genes up-regulated by NAA in wild type plants but down-regulated by NAA in *nsra/b* is significantly enriched for genes belonging to GO categories such as “response to hormone” (FDR < 1e-6); “response to water deprivation” (FDR < 5e-9). On the other hand cluster 3 genes (**Figure 6C**), e.g., down-regulated or not affected by NAA in wild type but up-regulated in the mutant are highly significantly enriched for GO categories related to pathogen responses such as “response to biotic stimulus” (FDR < 5e-16); “response to chitin” (FDR < 1e-26). We then confirmed the results of RNA-seq datasets (**Figure 7**) by RT-qPCR analysis of a small subset of genes belonging to clusters 2 and 3.

Given the important effect of NSRs on AS regulation, we also examined the putative function of differentially spliced genes having a switch in isoform usage. Strikingly, we identified a number of AS proteins located upstream of the immune response pathway. They include the MKP2 phosphatase (Lumbreras et al., 2010), the Toll/interleukin receptor (TIR) domain-containing protein TN1 and three members of the jasmonate co-receptor family (JAZ7, JAZ6, and JAZ2). In agreement, GO enrichment analysis of genes predicted to have significant isoforms switching events between *nsra/b* and Col-0 revealed a strong enrichment toward biological functions related to biotic stress responses (**Figure 6D**).

### NSRa Directly Recognizes Transcripts Involved in Biotic Stress Responses

**FIGURE 8:**
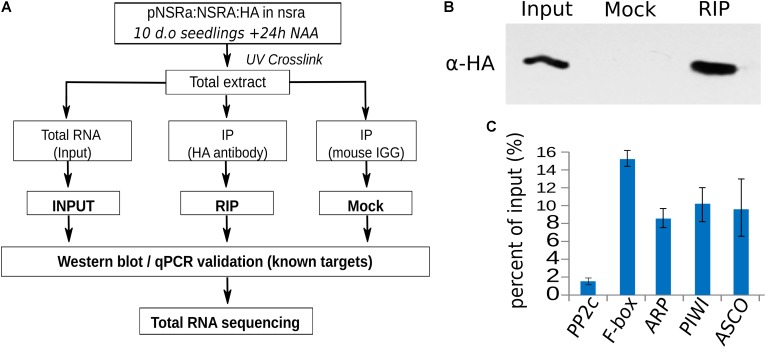
RNA immunoprecipitation of NSRa. **(A)** Experimental design to identify NSRa direct targets using RNA-immunoprecipitation assay. **(B)** Specificity of the immunoprecipitation demonstrated by a Western blot showing a discrete band at 27 kDa in the input and the RIP fraction but not the Mock IP (IgG) fraction. The membrane was blotted with HA antibody. **(C)** RT-qPCR analysis of previously identified (Bardou et al., 2014) NSRa targets (*FBOX, ARP, PIWI, ASCO*) and randomly selected abundant housekeeping genes (*PP2C*), showing the efficiency of the RIP assay toward target mRNAs.

**FIGURE 9:**
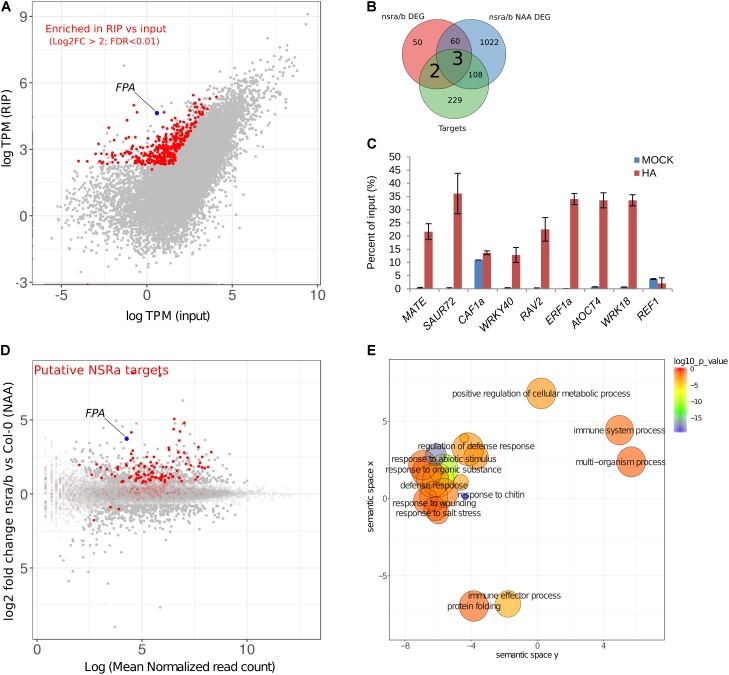
Identification of putative NSRa targets by RIP-seq. **(A)** Identification of NSRa targets: comparison of mean transcript abundance (TPM) in input vs. RIP-seq libraries Dots in red correspond to putative targets, e.g., significantly enriched transcripts in RIP as compared to input (FDR < 0.01 Log2 fold change > 2) and depleted in Mock IP. **(B)** Overlap between putative target genes and differentially regulated genes in *nsra/b* in mock (*nsra/b* DEG) or NAA-treated (*nsra/b* NAA DEG) seedlings. **(C)** RIP-qPCR assays using ProNSRa::NSRa::HA (NSRa) plants on total cell lysates of 10-day-old seedlings treated with 10 mM NAA for 24 h. Genes were randomly selected from NSRa putative target list Results of RT-qPCR are expressed as the mean of the percentage of input of three independent experiments ± standard error. **(D)** MA plot of showing the relationship between foldchange and transcript abundance for the comparison between *nsra/b* and Col-0 in the presence of NAA. Red dots correspond to putative NSRa targets. Plain dots correspond to differentially expressed genes. **(E)** REVIGO plots of GO enrichment clusters of putative target genes Each circle represents a significant GO category but only clusters with highest significance are labeled. Related GOs have similar (x, y) coordinates.

To address the question whether these targets are directly related to NSR function and/or indirectly affected by other proteins, we aimed to identify direct targets of NSRs using a genome-wide RIP-seq approach. We focused our analysis on NSRa as it is globally more highly expressed than NSRb (Bardou et al., 2014). Transgenic lines expressing an epitope tagged version of NSRa under its native promoter in the *nsra* mutant genetic background were used to avoid interference with the endogenous version of NSRa. Ten days-old seedlings treated for 24 h with NAA were used to match the transcriptome analysis. Immunoprecipitation was performed on UV cross-linked tissue using HA antibodies and mouse IgG as negative control (**Figure 8A**). NSRa-HA was detected from the input sample as well as from the eluate of the immunoprecipitation when it was performed with an HA antibody but not when mouse IgG were used (**Figure 8B**) qRT-PCR analysis of previously identified targets and a randomly selected abundant housekeeping gene confirmed the specific enrichment of target genes in the RIP sample compared to the input (**Figure 8C**). In addition, RNA extracted from mock IP eluate did not give detectable amount of RNA supporting the specificity of this assay. Total RNA-seq libraries were prepared in duplicate from input, RIP and Mock samples. PCA and correlation analysis showed a dispersion of the data compatible with statistical comparisons between groups (**Supplementary Figure S3**). To detect putative NSRa targets, we used a multi-factor differential expression analysis using DEseq2 in order to identify transcripts significantly enriched in RIP as compared to the input (FDR < 0.01; log2FC > 2) that were depleted from Mock samples. After filtering out all transcripts with less than two TPM in RIP libraries, we finally identified 342 putative targets of NSRa (**Figure 9A**).

Comparing this list of genes with those differentially expressed in *nsra/b* in mock or NAA treated seedling, we found that 33% of putative target genes were also deregulated in *nsra/b* (**Figure 9B**). Further examination of putative targets genes revealed that the large majority of these genes are up-regulated in *nsra/b* suggesting that NSRs are negatively controlling their transcript abundance *in vivo* (**Figure 9D**). GO enrichment analysis revealed that putative NSRa targets (**Figure 9E**) are enriched for genes involved in biological processes associated with defense responses such as “response to chitin” (FDR < 1.76e-9), “response to wounding” (FDR < 2.6e-3) or “immune system processes” (FDR < 1.7e-3). Interestingly, NSR target genes were also enriched for the GO category “regulation of transcription, DNA-templated” (FDR < 1.6e-8). Further examination of targets genes belonging to this GO category revealed that 56 transcription factors (TFs) are likely to be direct targets of NSRa (**Supplementary Table S1E**). Among them, we found the mRNA encoding the MYC2 TF, a key regulator of immune responses (Kazan and Manners, 2013) as well as nine *WRKY* and seven *ERF* TF transcripts, which both classes have been associated with the regulation of the plant immune response (Pandey and Somssich, 2009; Huang et al., 2016). Ten putative target genes were selected for RT-qPCR validation of the RIP assay. Among them, seven showed a significant enrichment over the input samples (**Figure 9C**) further supporting the genome-wide approach of NSRa target identification. Together, these results suggest that direct recognition of a subset of defense response genes by NSRa may affect their steady state abundance during auxin response.

### LncRNAs Are Overrepresented Among NSRa Targets

**FIGURE 10:**
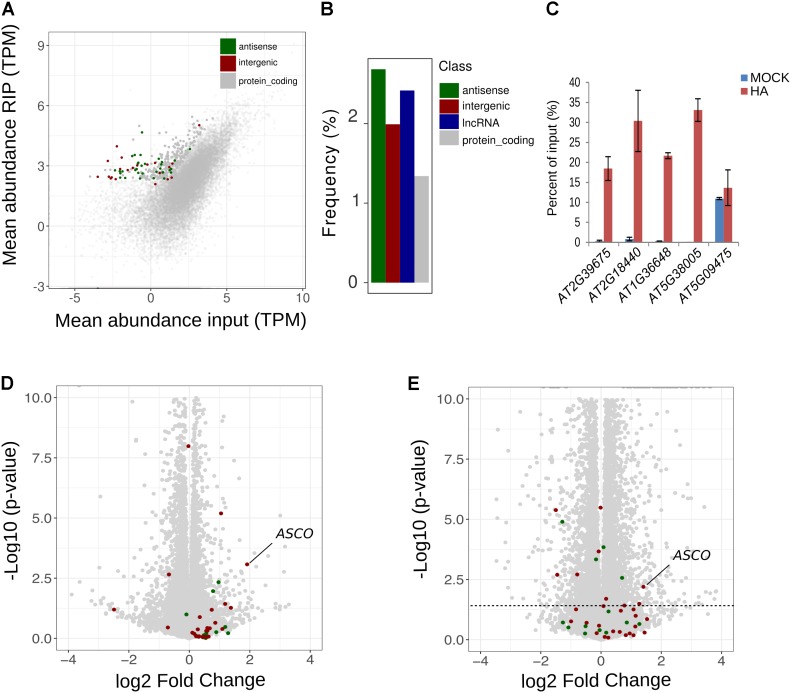
NSRa binds to numerous lncRNA. **(A)** Comparison of mean transcript abundance (TPM) in input vs. RIP-seq libraries. Dots in gray; red and green correspond to protein coding, intergenic and antisense lncRNA transcripts, respectively. Plain dots correspond to significantly enriched genes in RIP vs. Input, e.g., putative targets. **(B)** Frequency of all lncRNAs, antisense lncRNA, intergenic lncRNA andprotein coding genes among the NSRa targets: blue red, green, and gray bars, respectively. Frequency was calculated compared the number detected genes in the input for each class. **(C)** RIP-qPCR assays using proNSRa::NSRa::HA (NSRa) plants on total cell lysates of 10-day-old seedlings treated with 10mM NAA for 24 h. lncRNA were randomly selected from NSRa putative target list. Results of RT-qPCR are expressed as the mean of the percentage of input of three independent experiments ± standard error. Volcano plots of showing the relationship between the fold change and *p*-value of the comparison between *nsra/b* and Col-0 in **(D)** mock or **(E)** NAA treated samples. Plain colored dots correspond to intergenic (red) and antisense (blue) lncRNA which are putative targets of NSRa. The dotted line delineates a *p*-value of 0.05.

It was previously demonstrated that a direct interaction between NSR and the lncRNA *ASCO* is able to modulate NSR function (Bardou et al., 2014). Thus, we thoroughly analyzed global lncRNA abundance in RIP-seq datasets. Interestingly, lncRNAs appeared among the most highly enriched transcripts within the putative targets of NSRa. We found that, out of the 342 putative NSRa targets, 53 were lncRNA including 20 and 33 intergenic and antisense lncRNA, respectively (**Figure 10A**). In fact, relatively to the total number of lncRNAs detected in the input, lncRNA were significantly enriched over mRNA in the set of putative targets transcripts (hypergeometric test: 1.9 fold, p.value < 4.06e-4) (**Figure 10B**).

We further validated the NSR-lncRNA interaction by RIP-qPCR. We found four out of five lncRNA enriched over the input RNA in NSRa RIP samples (**Figure 10C**). Analyses of target lncRNA expression in *nsra/b* revealed that, similarly to the behavior of *ASCO*, seven target lncRNA are significantly upregulated in the *nsra/b* mutant (**Figures 10D,E**). Together, these results suggest that lncRNAs are overrepresented among targets of NSRa and that NSRs might control the accumulation of lncRNA *in vivo.* Future works on the interplay between lncRNA and mRNAs in NSR-containing complexes should shed light on their global impact over the transcriptome.

## Discussion

In agreement with our previous study based on microarrays, a novel thorough analysis of *nsra/b* transcriptome using RNA-seq has revealed an important role of these RBPs in the control of auxin-responsive genes. A previous study monitoring AS changes of a subset of 288 genes using high-resolution real-time PCR, first uncovered the important roles of NSR in auxin-driven AS changes and targeted RIP-qPCR showed that both NSR proteins were able to bind AS mRNA targets *in planta* (Bardou et al., 2014). Our global AS analysis further confirmed this function of NSRs on AS modulation and demonstrated the impact of these proteins at genome-wide level. However, our RIP-seq global analysis of NSR targets did not show a strong enrichment toward AS modulated transcripts. Instead, a large fraction of NSR targets were transcriptionally upregulated in *nsra/b*, suggesting that NSR may play a direct role in controlling their stability or transcription. Several splicing factors have been shown to affect transcription by interacting with the transcriptional machinery and to modulate Pol II elongation rates (Kornblihtt et al., 2004). In addition, specific RBPs deposited during pre-mRNA splicing at exon–exon splicing junctions, can influence their mRNA decay (Lumbreras et al., 2010; Nishtala et al., 2016). Further dissection of the NSR recognition sites on mRNAs may support a role of NSRs on mRNA decay.

The combination of our RNA-seq and RIP-seq approaches revealed that lncRNAs are privileged targets of NSRa and that a significant fraction of the auxin-responsive non-coding transcriptome is deregulated in the *nsra/b* genetic background. This is in accordance with our previous results showing that the specific interaction of NSR with the *ASCO* lncRNA is able to modify AS pattern of a subset of NSR-target genes. Our study suggests that NSRs may play a broader role in lncRNA biology. In particular, we found that a large majority of lncRNA targeted by NSRa are upregulated in *nsra/b*, suggesting a new role of these proteins in the control of lncRNA transcription and/or stability. So far, very little is known about lncRNA biogenesis, especially in plants. Other RBPs have been shown to affect lncRNA abundance. For instance several members of the cap binding complex such as CBP20, CBP80, and SERRATE have been shown to co-regulate the abundance of a large subset of lncRNAs in Arabidopsis seedlings (Liu et al., 2012). Interestingly, these three proteins, like NSRs, have also been associated with major roles in the control of AS patterns (Raczynska et al., 2010, 2014). This suggests that the splicing machinery might be used to control lncRNAs abundance in the nucleus and that the interplay between lncRNA and mRNAs may be an emerging mechanism in splicing regulation. Further genetic dissection is required to determine whether NSRs are involved in the same pathway that CBP20, CPB80, and SERRATE.

The strong deregulation of the *FLC/COOLAIR* module in *nsra/b* led us to identify a new role of NSRa in the control of flowering time. A number of forward genetic screenings aiming to identify new genes controlling flowering time through FLC expression modulation have consistently identified RNA processing and splicing factors that promote formation of the short *COOLAIR* isoforms, such as *FCA*, *FPA*, *HLP1*, *GRP7* and the core spliceosome component *PRP8a* (Deng and Cao, 2017). Loss of function mutants of these factors lead to a reduced usage of *COOLAIR* proximal polyadenylation site and an increase of *FLC* transcription which is associated with late flowering phenotypes (Deng and Cao, 2017). Interestingly, our analysis of the *FLC/COOLAIR* module in *nsr* mutants revealed an opposite role of NSRa in *COOLAIR* polyadenylation site usage, leading to the increased use of *COOLAIR* proximal polyadenylation site, and reduced *FLC* levels associated with an early flowering phenotype.

We also identified a new role of NSRs in the regulation of auxin-mediated expression and AS of transcripts related to biotic stress response. Interestingly, it has been shown for several years that natural (i.e., IAA) and synthetic (i.e., NAA) auxins can promote pathogen virulence of *P*. *syringae* (Mutka et al., 2013). More recently, a conserved pathway of auxin biosynthesis was demonstrated in *Pseudomonads* as contributing to pathogen virulence in *Arabidopsis thaliana* (McClerklin et al., 2018). However, little is known on the specific plant factors that modulate immune responses upon endogenous or pathogen produced auxins. Our work shows that NSRs do not affect the global auxin responses but rather have an impact on the abundance of mRNAs coding for proteins involved in plant immune response, suggesting that these RBPs may participate in the regulation of plant defense by endogenous or pathogen-produced auxins.

In higher plants, AS plays a key role in gene expression as shown by the fact that 60–70% of intron-containing genes undergoes alternative processing. Several genome-wide studies of AS has shown that this mechanism may represent a way to enhance the ability for plant cells to cope with stress via the modulation of transcriptome plasticity. Here we show that among the genes with significant isoforms switching events in *nsra/b* mutant treated with auxin, we identified several genes involved in the modulation of the MAPK kinase modules, a core regulator of defense responses. They included MKP2 phosphatase which functionally interacts with MPK3 and MPK6 to mediate disease response in Arabidopsis (Lumbreras et al., 2010) and PTI-4 kinase which was found in MPK6 containing complexes *in vivo* and was shown to function in the MPK6 signaling cascade (Forzani et al., 2011). As activation of MAPK signaling cascades regulate the expression of 1000s of downstream targets genes, we can speculate that a large fraction of the transcriptome change observed in *nsra/b* mutant could be a consequence of AS defect of genes involved in such early phase of the defense response pathway.

## Materials and Methods

### Plant Material and Treatments

All mutants were in the Columbia-0 (Col-0) background. *Atnsra* (SALK_003214) and *Atnsrb* (Sail_717) were from the SALK and SAIL T-DNA collections, respectively. For RIP, a lines expressing pNSRa::NRSa-HA in *Atnsra* or pNSRa::NRSb-HA in *Atnsrb* were used (Bardou et al., 2014). Plants were grown on soil in long day (16 h light/8 h dark) conditions at 23°C. For RNA-sequencing and RIP-seq WT and *nsra/nsrb* were grown on nylon membrane (Nitex 100 μm) in plate filled with ½MS medium for 10 days and then transferred for 24 h to ½MS medium containing 100 nM NAA or a mock solution before the whole seedlings were harvested. For flowering time analysis, plants were grown under long day conditions and the number of rosette leave were counted from 12 plants when the flower stem was 1 cm tall.

### RNA Sequencing Analysis

Stranded mRNA sequencing libraries were performed on three biological replicate of Col-0,*nsra/b* treated with a 100 nM NAA or a mock solution. One μg of total RNA from Col-0 and *nsra/b* seedlings was used for library preparation using the Illumina TruSeq Stranded mRNA library prep kit according to the manufacturer instruction. Libraries were sequenced on an HiSeq2000 sequencer using 150 nt pair-end read mode. A minimum 28 Million of were obtained for each sample, quality filtered using fastqc (Andrews, 2010) with default parameters and aligned using tophat (Trapnell et al., 2012) with the following arguments: -g 1 -i 5 -p 6 -I 2000 –segment-mismatches 2 –segment-length 20 –library-type fr-firststrand. Read were counted using SummarizeOverlap function from the GenomicRange R package (Lawrence et al., 2013) using strand specific and Union mode. Differential gene expression analysis was done one pairwise comparison using DEseq2 (Love et al., 2014) with FDR correction of the *p*-value. K-mean clustering analysis was performed in R on scaled log2 fold change data and the optimal number of cluster was determined using the elbow method. Heatmap was plotted using heatmap.2 function of the gplots package (Warnes et al., 2009). Sequence files have been submitted to the NCBI GEO database under accession GSE65717 and GSE116923.

### Gene Ontology Analysis

Gene ontology enrichment analysis was done using the AgriGO server^1^ using default parameters. Lists of GO terms were visualized using REVIGO^2^ and plotted in R. Only GO terms with a dispensability factor over 0.5 were printed in REVIGO plots.

### AS Analysis

RNAprof (v1.2.6) was used on BAM alignment files with the following parameters: LIBTYPE = fr-unstranded, SEQTYPE = “–Pair”, MIS = 1000. All possible pairwise comparisons were computed. Overlap of differential events (pval < 1e-04) with gene annotation was done using *findOverlaps* of the GenomicRanges Package in R and custom in house scripts. Only events that were completely included in gene feature (e.g., intron, exons, 3′ UTR, and 5′ UTR) were kept for further analysis.

For isoforms switching identification, transcript isoforms abundance was quantified with pseudo alignment read count with *kallisto* (Bray et al., 2016), on all isoforms of the AtRTD2 database (Zhang et al., 2017). Then the IsoformSwitchAnalyzeR package was used to detect significant changes in isoform usage. Only significant switches (p.adj < 0.1) were kept for further analyses (Vitting-Seerup and Sandelin, 2017).

### RNA Immunoprecipitation and Sequencing (RIP-Seq)

NSRa protein tagged with HA was immunoprecipitated from the *nrsa* mutant background expressing the pNSRa:NSRa-HA construct (Bardou et al., 2014). Briefly, 10 day old seedlings treated with 100 nM NAA for 24 h were irradiated three times with UV using a UV crosslinker CL-508 (Uvitec) at 0.400 J/cm^2^. Plants were ground in liquid nitrogen and RNA-IP was performed as in Sorenson and Bailey-Serres (2014) with the following modification: immunoprecipitation (IP) was performed using anti mouse HA-7 monoclonal antibody (Sigma) and the negative IP (Mock) was done using anti mouse IgG (Millipore). RNA was eluted from the beads with 50 U proteinase K (RNase grade, Invitrogen) in 2 μl of RNase inhibitor at 55°C for 1 h in wash buffer and extracted using Trizol according to manufacturer instructions. A 10th of the input fraction was saved for RNA and protein extraction. For western blot analysis, proteins were extracted from the beads and input fraction with 2X SDS-loading Buffer for 10 min at 75°C, directly loaded on SDS PAGE, transferred onto Nitrocellulose membranes and blotted with HA-7 antibody. For RT-qPCR analysis, RNA was reverse transcribed with Maxima Reverse Transcriptase (Thermo) using random Hexamer priming. cDNA from input, IP and Mock were amplified with primers listed in **Supplementary Table S2**. Results were analyzed using the percentage of input method. First, Ct values of input sample (10% of volume) were adjusted to 100% as follows: Adjusted Ct input = Raw Ct input-log2(10). Percentage of input was calculated as follow: 100^∗^2ˆ(Adjusted Ct input - Ct IP). Results are mean of three independent experiments. Student’s *t*-test was performed to determine significance. For RNA-seq : input mock and IP RNA were depleted of rRNA using the plant leaf ribozero kit (Illumina) and libraries were prepared using the Illumina TruSeq Stranded mRNA library prep kit according to the manufacturer instruction but omitting the polyA RNA purification step and sequenced on a NextSeq500 sequencer (Illumina) using single-end 75 bp reads mode. Sequence files have been submitted to the NCBI GEO database under accession GSE116914.

### Analysis of RIP-Seq Data

Reads were mapped using STAR (Dobin et al., 2013) and TPM was calculated using RSEM (Li and Dewey, 2011). Read were counted using SummarizeOverlap function from the GenomicRange R package (Lawrence et al., 2013) using strand specific and *Union* mode. To identify putative NSRa targets we used pairwise comparison with DESeq2 package. Only genes significantly enriched in IP with anti HA as compared with the anti-mouse IgG (mock) IP were kept for further analysis (logFC > = 1; FDR < 0.01). Putative targets genes were defined as gene highly enriched in the IP with anti HA compared to their global level in input used for the IP (logFC > 2; FDR < 0.01). To reduce noise associated with low read counts, we excluded from this list any gene with less than two TPM in at least one of the RIP-seq libraries.

### Measuring Distal and Proximal *COOLAIR Variants*

This was performed essentially as in Marquardt et al. (2014). 5 μg of total RNA was reverse transcribed with and oligo(dT) primer. qPCR was performed with set of primers specific to distal and proximal *COOLAIR* described in Marquardt et al. (2014). qPCR reactions were performed in triplicates for each sample. Average values of the triplicates were normalized to the expression of total *COOLAIR* quantified in the same sample.

## Author Contributions

JB designed study, performed the experiments, analyzed the data, and wrote the article. NR, FA, RR, and CC performed the experiments and participated in writing. TB analyzed the data. MC designed the study and wrote the paper.

## Conflict of Interest Statement

The authors declare that the research was conducted in the absence of any commercial or financial relationships that could be construed as a potential conflict of interest.

## Footnotes

1 http://bioinfo.cau.edu.cn/agriGO/

2 http://revigo.irb.hr/

## Supplementary Material

FIGURE S1 **(A)** Pearson correlation matrix heatmap with dendograms showing the relative distance between each poly(A)+ RNA-seq samples. **(B)** PCA analysis showing the effect of auxin and genotype on the variance between samples.

FIGURE S2 **(A)** Proximal and **(B)** distal variant relative abundance normalized to an housekeeping transcript (PP2C). Error bars correspond to ± the standard deviation of three biological replicates. Significance was determined using a Student’s *t*-test (^∗∗∗^*p*-value < 0.001).

FIGURE S3 **(A)** Pearson correlation matrix heatmap with dendograms showing the relative distance between each sample of the RIP-seq experiments. **(B)** PCA analysis showing the effect the variance between samples.

TABLE S1 Summary of RNA-seq and RIP-seq data analysis. **(A)** Description of spreadsheet tab. **(B)** Differential gene expression analysis. **(C)** RNA prof analysis. **(D)** Expression and usage of all isoforms from genes containing at least one isoforms switching event. **(E)** NSRa targets identified by RIP. **(F)** Transcription Factor identified in NSRa targets.

TABLE S2 Sequence of primers used in this study.

## References

[B1] Andrews S. (2010). *FastQC: A Quality Control Tool for High Throughput Sequence Data*. Available at: http://www.bioinformatics.babraham.ac.uk/projects/fastqc

[B2] Ariel F. Jegu T. Latrasse D. Romero-Barrios N. Christ A. Benhamed M. (2014). Noncoding transcription by alternative RNA polymerases dynamically regulates an auxin-driven chromatin loop. *Mol. Cell* 55 383–396. 10.1016/j.molcel.2014.06.011 25018019

[B3] Bailey-Serres J. Sorenson R. Juntawong P. (2009). Getting the message across: cytoplasmic ribonucleoprotein complexes. *Trends Plant Sci.* 14 443–453. 10.1016/j.tplants.2009.05.004 19616989

[B4] Bardou F. Ariel F. Simpson C. G. Romero-Barrios N. Laporte P. Balzergue S. (2014). Long noncoding RNA modulates alternative splicing regulators in *Arabidopsis*. *Dev. Cell* 30 166–176. 10.1016/j.devcel.2014.06.017 25073154

[B5] Bray N. L. Pimentel H. Melsted P. Pachter L. (2016). Near-optimal probabilistic RNA-seq quantification. *Nat. Biotechnol.* 34 525–527. 10.1038/nbt.3519 27043002

[B6] Brown J. W. Calixto C. P. Zhang R. (2017). High-quality reference transcript datasets hold the key to transcript-specific RNA-sequencing analysis in plants. *New Phytol.* 213 525–530. 10.1111/nph.14208 27659901

[B7] Deng X. Cao X. (2017). Roles of pre-mRNA splicing and polyadenylation in plant development. *Curr. Opin. Plant Biol.* 35 45–53. 10.1016/j.pbi.2016.11.003 27866125

[B8] Dobin A. Davis C. A. Schlesinger F. Drenkow J. Zaleski C. Jha S. (2013). STAR: ultrafast universal RNA-seq aligner. *Bioinformatics* 29 15–21. 10.1093/bioinformatics/bts635 23104886 PMC3530905

[B9] Foley S. W. Kramer M. C. Gregory B. D. (2017). RNA structure, binding, and coordination in *Arabidopsis*. *Wiley Interdiscip. Rev. RNA* 8:e1426. 10.1002/wrna.1426 28660659

[B10] Forzani C. Carreri A. de la Fuente van Bentem S. Lecourieux D. Lecourieux F. Hirt H. (2011). The *Arabidopsis* protein kinase Pto-interacting 1-4 is a common target of the oxidative signal-inducible 1 and mitogen-activated protein kinases. *FEBS J.* 278 1126–1136. 10.1111/j.1742-4658.2011.08033.x 21276203

[B11] Gagliardi M. Matarazzo M. R. (2016). RIP: RNA immunoprecipitation. *Methods Mol. Biol.* 1480 73–86. 27659976 10.1007/978-1-4939-6380-5_7

[B12] Hornyik C. Terzi L. C. Simpson G. G. (2010). The spen family protein FPA controls alternative cleavage and polyadenylation of RNA. *Dev. Cell* 18 203–213. 10.1016/j.devcel.2009.12.009 20079695

[B13] Huang P.-Y. Catinot J. Zimmerli L. (2016). Ethylene response factors in *Arabidopsis* immunity. *J. Exp. Bot.* 67 1231–1241. 10.1093/jxb/erv518 26663391

[B14] Kazan K. Manners J. M. (2013). MYC2: the master in action. *Mol. Plant* 6 686–703. 10.1093/mp/sss128 23142764

[B15] Kornblihtt A. R. de la Mata M. Fededa J. P. Munoz M. J. Nogues G. (2004). Multiple links between transcription and splicing. *RNA* 10 1489–1498. 15383674 10.1261/rna.7100104 PMC1370635

[B16] Köster T. Meyer K. (2018). Plant ribonomics: proteins in search of RNA partners. *Trends Plant Sci.* 23 352–365. 10.1016/j.tplants.2018.01.004 29429586

[B17] Lawrence M. Huber W. Pagès H. Aboyoun P. Carlson M. Gentleman R. (2013). Software for computing and annotating genomic ranges. *PLoS Comput. Biol.* 9:e1003118. 10.1371/journal.pcbi.1003118 23950696 PMC3738458

[B18] Lee K. Kang H. (2016). Emerging roles of RNA-binding proteins in plant growth, development, and stress responses. *Mol. Cells* 39 179–185. 10.14348/molcells.2016.2359 26831454 PMC4794599

[B19] Li B. Dewey C. N. (2011). RSEM: accurate transcript quantification from RNA-seq data with or without a reference genome. *BMC Bioinformatics* 12:323. 10.1186/1471-2105-12-323 21816040 PMC3163565

[B20] Liu F. Marquardt S. Lister C. Swiezewski S. Dean C. (2009). Targeted 3′ processing of antisense transcripts triggers *Arabidopsis* FLC chromatin silencing. *Science* 327 94–97. 19965720 10.1126/science.1180278

[B21] Liu J. Jung C. Xu J. Wang H. Deng S. Bernad L. (2012). Genome-wide analysis uncovers regulation of long intergenic noncoding RNAs in *Arabidopsis*. *Plant Cell* 24 4333–4345. 10.1105/tpc.112.102855 23136377 PMC3531837

[B22] Love M. I. Huber W. Anders S. (2014). Moderated estimation of fold change and dispersion for RNA-seq data with DESeq2. *Genome Biol.* 15:550. 10.1101/002832 25516281 PMC4302049

[B23] Lumbreras V. Vilela B. Irar S. Solé M. Capellades M. Valls M. (2010). MAPK phosphatase MKP2 mediates disease responses in *Arabidopsis* and functionally interacts with MPK3 and MPK6. *Plant J.* 63 1017–1030. 10.1111/j.1365-313X.2010.04297.x 20626661

[B24] Maquat L. E. (2004). Nonsense-mediated mRNA decay: splicing, translation and mRNP dynamics. *Nat. Rev. Mol. Cell Biol.* 5 89–99. 15040442 10.1038/nrm1310

[B25] Marondedze C. Thomas L. Serrano N. L. Lilley K. S. Gehring C. (2016). The RNA-binding protein repertoire of *Arabidopsis thaliana*. *Sci. Rep.* 6:29766. 10.1038/srep29766 27405932 PMC4942612

[B26] Marquardt S. Raitskin O. Wu Z. Liu F. Sun Q. Dean C. (2014). Functional consequences of splicing of the antisense transcript COOLAIR on FLC transcription. *Mol. Cell* 54 156–165. 10.1016/j.molcel.2014.03.026 24725596 PMC3988885

[B27] McClerklin S. A. Lee S. G. Harper C. P. Nwumeh R. Jez J. M. Kunkel B. N. (2018). Indole-3-acetaldehyde dehydrogenase-dependent auxin synthesis contributes to virulence of *Pseudomonas syringae* strain DC3000. *PLoS Pathog.* 14:e1006811. 10.1371/journal.ppat.1006811 29293681 PMC5766252

[B28] Mutka A. M. Fawley S. Tsao T. Kunkel B. N. (2013). Auxin promotes susceptibility to *Pseudomonas syringae* via a mechanism independent of suppression of salicylic acid-mediated defenses. *Plant J.* 74 746–754. 10.1111/tpj.12157 23521356

[B29] Ner-Gaon H. Halachmi R. Savaldi-Goldstein S. Rubin E. Ophir R. Fluhr R. (2004). Intron retention is a major phenomenon in alternative splicing in *Arabidopsis*. *Plant J.* 39 877–885. 15341630 10.1111/j.1365-313X.2004.02172.x

[B30] Nishtala S. Neelamraju Y. Janga S. C. (2016). Dissecting the expression relationships between RNA-binding proteins and their cognate targets in eukaryotic post-transcriptional regulatory networks. *Sci. Rep.* 6:25711. 10.1038/srep25711 27161996 PMC4861959

[B31] Oliveira C. Faoro H. Alves L. R. Goldenberg S. (2017). RNA-binding proteins and their role in the regulation of gene expression in *Trypanosoma cruzi* and *Saccharomyces cerevisiae*. *Genet. Mol. Biol.* 40 22–30. 10.1590/1678-4685-GMB-2016-0258 28463381 PMC5409782

[B32] Pandey S. P. Somssich I. E. (2009). The role of WRKY transcription factors in plant immunity. *Plant Physiol.* 150 1648–1655. 19420325 10.1104/pp.109.138990 PMC2719123

[B33] Raczynska K. D. Simpson C. G. Ciesiolka A. Szewc L. Lewandowska D. McNicol J. (2010). Involvement of the nuclear cap-binding protein complex in alternative splicing in *Arabidopsis thaliana*. *Nucleic Acids Res.* 38 265–278. 10.1093/nar/gkp869 19864257 PMC2800227

[B34] Raczynska K. D. Stepien A. Kierzkowski D. Kalak M. Bajczyk M. McNicol J. (2014). The SERRATE protein is involved in alternative splicing in *Arabidopsis thaliana*. *Nucleic Acids Res.* 42 1224–1244. 10.1093/nar/gkt894 24137006 PMC3902902

[B35] Romero-Barrios N. Legascue M. F. Benhamed M. Ariel F. Crespi M. (2018). Splicing regulation by long noncoding RNAs. *Nucleic Acids Res.* 46 2169–2184. 10.1093/nar/gky095 29425321 PMC5861421

[B36] Sorenson R. Bailey-Serres J. (2014). Selective mRNA sequestration by oligouridylate-binding protein 1 contributes to translational control during hypoxia in *Arabidopsis*. *Proc. Natl. Acad. Sci. U.S.A.* 111 2373–2378. 10.1073/pnas.1314851111 24469793 PMC3926019

[B37] Tran V. D. T. Souiai O. Romero-Barrios N. Crespi M. Gautheret D. (2016). Detection of generic differential RNA processing events from RNA-seq data. *RNA Biol.* 13 59–67. 10.1080/15476286.2015.1118604 26849165 PMC4829270

[B38] Trapnell C. Roberts A. Goff L. Pertea G. Kim D. Kelley D. R. (2012). Differential gene and transcript expression analysis of RNA-seq experiments with TopHat and Cufflinks. *Nat. Protoc.* 7 562–578. 10.1038/nprot.2012.016 22383036 PMC3334321

[B39] Vitting-Seerup K. Sandelin A. (2017). The landscape of isoform switches in human cancers. *Mol. Cancer Res.* 15 1206–1220. 10.1158/1541-7786.MCR-16-0459 28584021

[B40] Warnes G. R. Bolker B. Bonebakker L. Gentleman R. Huber W. Liaw A. (2009). *gplots: Various R programming Tools for Plotting Data. R Package Version 2*.

[B41] Whittaker C. Dean C. (2017). The FLC locus: a platform for discoveries in epigenetics and adaptation. *Annu. Rev. Cell Dev. Biol.* 33 555–575. 10.1146/annurev-cellbio-100616-060546 28693387

[B42] Zhang R. Calixto C. P. G. Marquez Y. Venhuizen P. Tzioutziou N. A. Guo W. (2017). A high quality *Arabidopsis* transcriptome for accurate transcript-level analysis of alternative splicing. *Nucleic Acids Res.* 45 5061–5073. 10.1093/nar/gkx267 28402429 PMC5435985

