# Supplementary figures and images for: Nuclear Speckle RNA Binding Proteins Remodel Alternative Splicing and the Non-coding Arabidopsis Transcriptome to Regulate a Cross-Talk Between Auxin and Immune Responses
Source: Front Plant Sci. 2018 Aug 21;9:1209. doi: 10.3389/fpls.2018.01209 (PMC6111844; doi:10.3389/fpls.2018.01209)

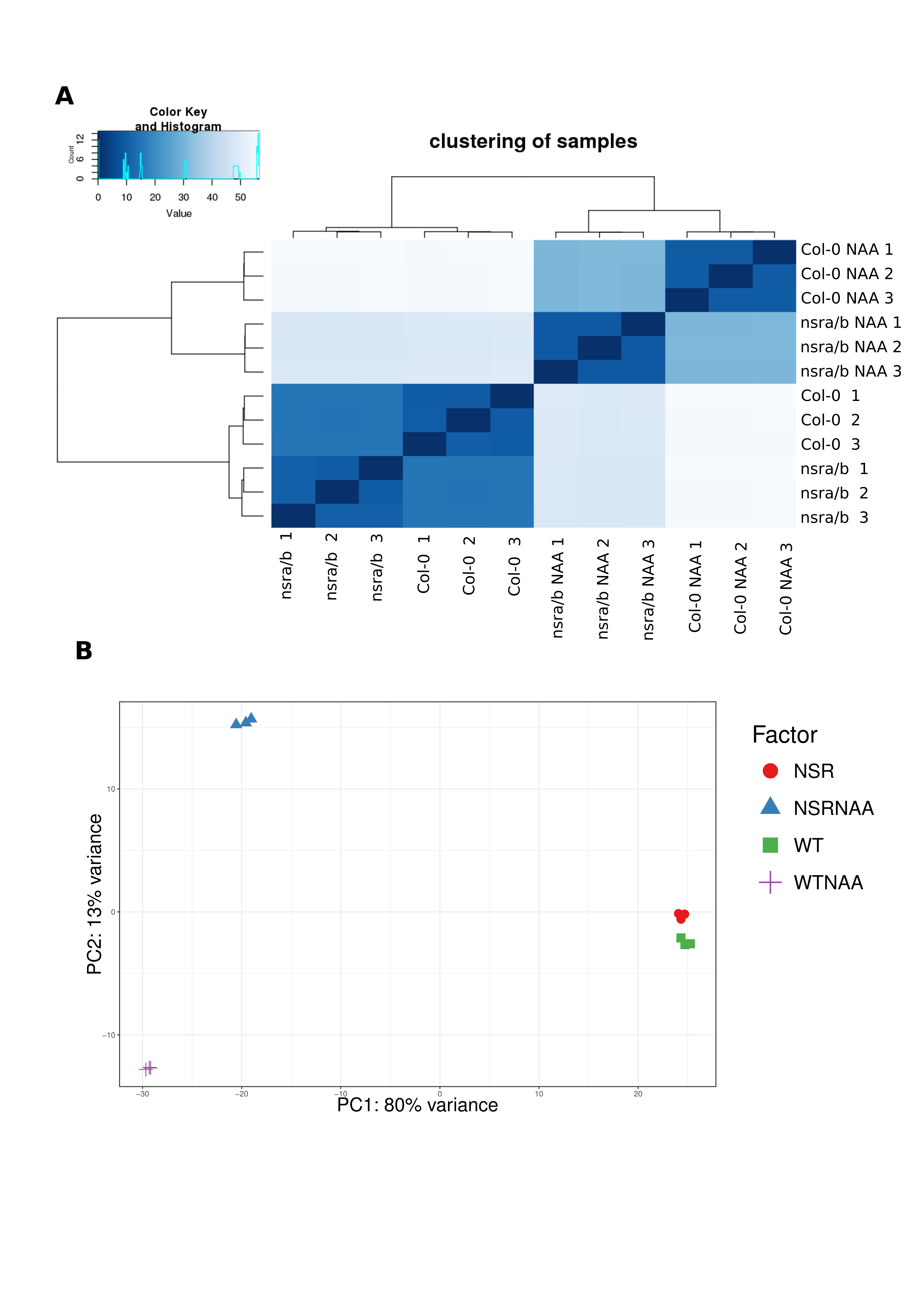

Supplement: FIGURE S1 — (A) Pearson correlation matrix heatmap with dendograms showing the relative distance between each poly(A)+ RNA-seq samples. (B) PCA analysis showing the effect of auxin and genotype on the variance between samples. [file Image_1.TIFF]

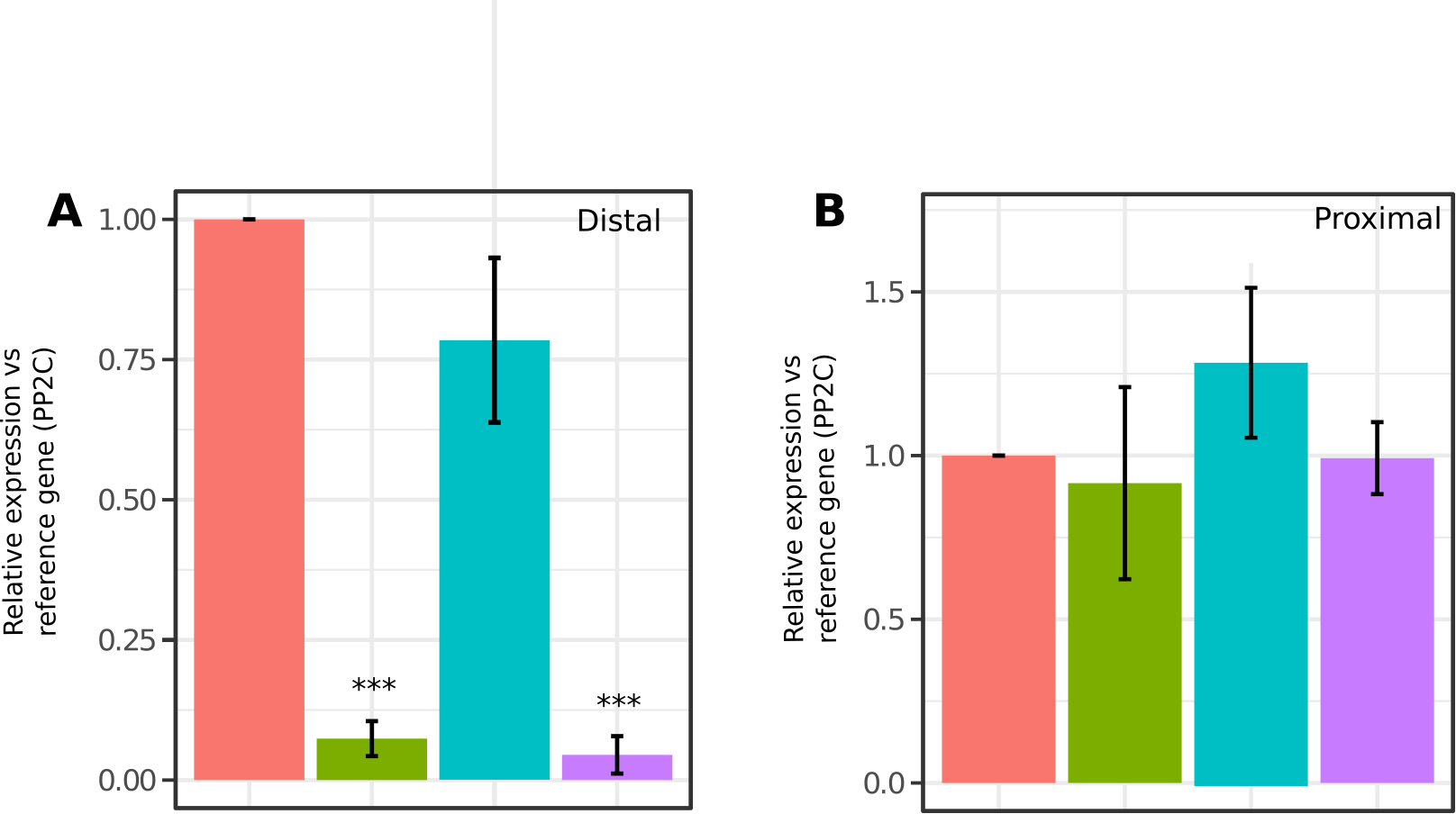

Supplement: FIGURE S2 — (A) Proximal and (B) distal variant relative abundance normalized to an housekeeping transcript (PP2C). Error bars correspond to ± the standard deviation of three biological replicates. Significance was determined using a Student’s t-test (∗∗∗p-value < 0.001). [file Image_2.tiff]

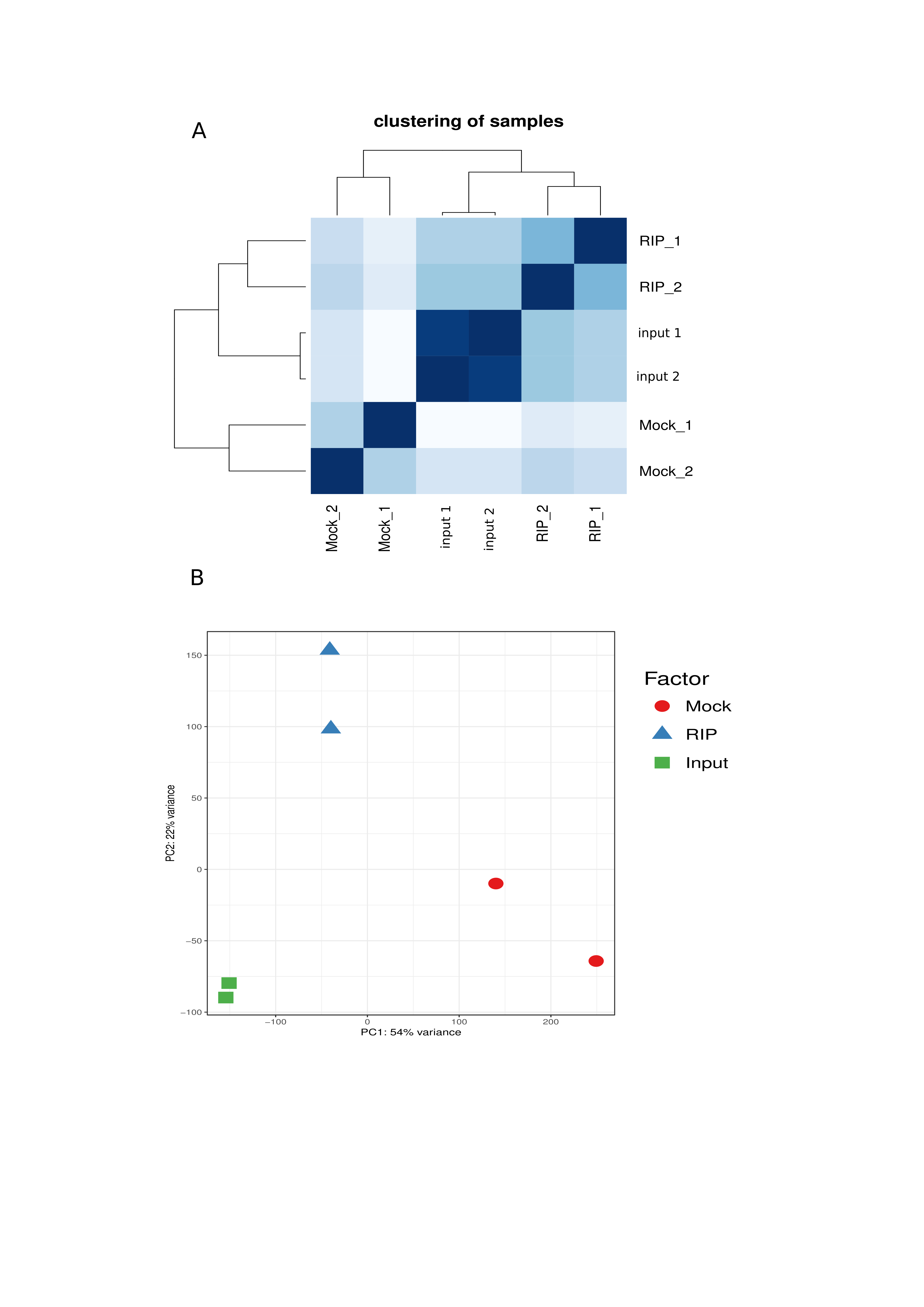

Supplement: FIGURE S3 — (A) Pearson correlation matrix heatmap with dendograms showing the relative distance between each sample of the RIP-seq experiments. (B) PCA analysis showing the effect the variance between samples. [file Image_3.TIFF]
