# Supplementary material for: Nuclear Speckle RNA Binding Proteins Remodel Alternative Splicing and the Non-coding Arabidopsis Transcriptome to Regulate a Cross-Talk Between Auxin and Immune Responses
Source: Front Plant Sci. 2018 Aug 21;9:1209. doi: 10.3389/fpls.2018.01209 (PMC6111844; doi:10.3389/fpls.2018.01209)
Supplement: TABLE S2 — Sequence of primers used in this study. [file Table_2.DOCX]

| Primer name | Gene name | orientation | sequence |
| --- | --- | --- | --- |
| 10073 | AT1G39675 | Fw | ACCATGACTCGATCTAACGGCGGA |
| 10072 | AT1G39675 | Rv | AGCGCCGTCAAGCTCTGCAA |
| 10071 | At2g18440 | Fw | CATGGCAAAGGTCGGATGACAGAG |
| 10070 | At2g18440 | Rv | GACGGATGTGTGTGTGTTGGATTGA |
| 10069 | AT4G36648 | Fw | TTGCCATGACAGGTGCGCTTGC |
| 10068 | AT4G36648 | Rv | AAGAGGGGAGAACCCGGGCAAA |
| 10878 | AT5G38005 | Fw | AAACCCATTGCTGCAACTGCTC |
| 10877 | AT5G38005 | Rv | TTGTGGGTTCGTCTGCTGTG |
| 10878 | AT5G09475 | Fw | TGCATACTCCAACGCAAACA |
| 10879 | AT5G09475 | Rv | GGGGAGACACTAGAACCACA |
| 10067 | SAUR72 | Fw | TCCCGTCACCGATTAGATCCTGAAC |
| 10066 | SAUR72 | Rv | TCGTTAGCGCGGAGCTACTTAACC |
| 10065 | CAF1a | Fw | CGCCCGGTTAACTTCAAGAGACC |
| 10064 | CAF1a | Rv | GTGGCGTTGCGTGAGTTTTTAGG |
| 10063 | RAV2 | Fw | CAAGCTGCGATGTCGTAGGAACG |
| 10062 | RAV2 | Rv | GACGGAGTCACGAAAGCTACCATCT |
| 10061 | ERF1 | Fw | AGACGAAGAAGACGGCTCCCAACC |
| 10060 | ERF1 | Rv | ACGGACGAAACCCTAGCTTTAGCA |
| 10059 | OCT4 | Fw | AAGCTGTCGCGATACCAAAGATTGC |
| 10058 | OCT4 | Rv | GTGTCGGAGTGGGGATTGATTTGC |
| 10057 | WRKY18 | Fw | TCTGCGCTGCGTTGTACCTTCTT |
| 10056 | WRKY18 | Rv | TTATGCTCCGCCGTGTAAGTGACG |
| 10834 | FLC | Fw | AAATGCTGAAAGAAGAGAACCAG |
| 10832 | FLC | Rv | TCTCCATCTCAGCTTCTGCTC |
| 9010 | FPA | Fw | TGACCTCTCATCGACACCCA |
| 9009 | FPA | Rv | CCTCCCATCTCCTGCACAAG |
| 11051 | AT2G00370 | Fw | AGCGAGAGAATGGTGAGTCGA |
| 11050 | AT2G00370 | Rv | CACCTTTGTAGAAGCTGCGTGT |
| 11049 | MATE | Fw | ATAGCCACCATTCTAGGCAAACC |
| 11048 | MATE | Rv | ATGTGAGGTACTCCAGCTCCTG |
| 11047 | SKIP23 | Fw | ACCCACAGTTCGTCGTAATCCG |
| 11046 | SKIP23 | Rv | TTATGCAGCACCTCGCCGTTTC |
| 11045 | THIONIN | Fw | CCCTCGAAGTCTCAATCGTAGGTG |
| 11044 | THIONIN | Rv | TTTCGTACACCTTCCGGAGTGC |
| 11043 | JAZ13 | Fw | ATTCCAGTCTCACCGGCACTAAC |
| 11042 | JAZ13 | Rv | TAGAGATGGCGAGCAAGGATCGTG |
| 11041 | TIR-NBS | Fw | TGTTTGCCTCATCGTCCCTGTC |
| 11040 | TIR-NBS | Rv | AATGGAGCCACGCTTTGACCTG |
| 11039 | GRP | Fw | ACCGATAAAGCCAACCAACAGACC |
| 11038 | GRP | Rv | CAAGCGGCAGCATGAATCTTCG |
| 10558 | WRKY40 | Fw | CCTCTCGGTTATGTTGCTCTTG |
| 10557 | WRKY40 | Rv | AAGATCCACCGACAAGTGC |
| 8272 | JAZ8 | Fw | CGTCGTGAATGGTACGGTGA |
| 8271 | JAZ8 | Rv | CTCAAACGGGTCGGATCCTC |
| 10878 | AT5G38005 | Fw | AAACCCATTGCTGCAACTGCTC |
| 10877 | AT5G38005 | Rv | TTGTGGGTTCGTCTGCTGTG |
| 10878 | AT5G09475 | Fw | TGCATACTCCAACGCAAACA |
| 10879 | AT5G09475 | Rv | GGGGAGACACTAGAACCACA |
